# Sestrin2 Suppresses Classically Activated Macrophages-Mediated Inflammatory Response in Myocardial Infarction through Inhibition of mTORC1 Signaling

**DOI:** 10.3389/fimmu.2017.00728

**Published:** 2017-06-30

**Authors:** Keping Yang, Chenhong Xu, Yunfeng Zhang, Shaolin He, Dazhu Li

**Affiliations:** ^1^Department of Cardiology, Jingzhou Central Hospital, Jingzhou Clinical Medical College, Yangtze University, Jingzhou, China; ^2^Department of Cardiology, Union Hospital, Tongji Medical College, Huazhong University of Science and Technology, Wuhan, China

**Keywords:** Sestrin2, macrophages, myocardial infarction, mTORC1, inflammation

## Abstract

Myocardial infarction (MI) triggers an intense inflammatory response that is essential for dead tissue clearance but also detrimental to cardiac repair. Macrophages are active and critical players in the inflammatory response after MI. Understanding the molecular mechanisms by which macrophage-mediated inflammatory response is regulated is important for designing new therapeutic interventions for MI. In the current study, we examined the role of Sestrin2, which is a stress-inducible protein that regulate metabolic homeostasis, in the regulation of inflammatory response of cardiac macrophages after MI. We found that cardiac macrophages upregulated Sestrin2 expression in a mouse MI model. Using a lentiviral transduction system to overexpress Sestrin2 in polarized M1 and M2 macrophages, we revealed that Sestrin2 predominantly functioned on M1 rather than M2 macrophages. Sestrin2 overexpression suppressed inflammatory response of M1 macrophages both *in vitro* and *in vivo*. Furthermore, in the mouse MI model with selective depletion of endogenous macrophages and adoptive transfer of exogenous Sestrin2-overexpressing macrophages, the anti-inflammatory and repair-promoting effect of Sestrin2-overexpressing macrophages was demonstrated. Furthermore, Sestrin2 significantly inhibited mTORC1 signaling in M1 macrophages. Taken together, our study indicates the importance of Sestrin2 for suppression of M1 macrophage-mediated cardiac inflammation after MI.

## Introduction

An inflammatory response, which is important for cardiac repair but also implicated in the progression of cardiac remodeling and heart failure, is triggered by myocardial infarction (MI) (1). A balanced inflammatory response as well as timely resolution of inflammation in the acute phase of MI might be a prerequisite of myocardial repair and regeneration. Therefore, targeting the inflammatory response could be an important therapeutic strategy for MI.

The post-MI inflammatory response includes substantial recruitment of circulating leukocytes into infarcted myocardium (2). Among these infiltrating leukocytes, monocytes/macrophages are active and critical players in the modulation of post-MI inflammatory response (3). It has been shown that both classically activated macrophages (pro-inflammatory M1) and alternatively activated macrophages (anti-inflammatory M2) are present in infarcted myocardium, probably reflecting differential activation during different phases of scar tissue formation after MI (4, 5). The significance of macrophages for the progression and resolution of infarct inflammation, as well as for the tissue healing and remodeling, has been suggested in previous studies using selective depletion of monocytes/macrophages (4, 6, 7). Due to their crucial role in the pathophysiological processes triggered by MI, monocytes/macrophages therefore represent a potential therapeutic target to promote myocardial repair and functional regeneration. However, the molecular mechanisms underlying the recruitment and functions of monocytes/macrophages upon the onset of MI have not been thoroughly elucidated.

Sestrins are a family of stress-inducible proteins that regulate metabolic homeostasis (8). Loss of endogenous Sestrins can provoke a variety of metabolic pathologies, including insulin resistance, fat accumulation, mitochondrial dysfunction, and oxidative damage (9, 10). Particularly, Sestrin2 is important for myocardial protection against ischemic damage, acting as an LKB1-AMPK scaffold for the initiation of AMPK signaling after ischemic stroke (11). In addition, in macrophage cell line RAW264.7, Sestrin2 remarkably inhibits lipopolysaccharide-induced NO release, iNOS expression, and pro-inflammatory cytokine production (12). Furthermore, Sestrin2 is a protective molecule against RAW264.7 cell apoptosis induced by oxidized low-density lipoprotein (13). However, whether Sestrin2 has the same functions in primary monocytes/macrophages, especially in cardiac macrophages after MI, has not been studied.

In this study, we examined the role of Sestrin2 in the regulation of inflammatory response of cardiac macrophages after MI. We found that cardiac macrophages upregulated Sestrin2 expression in a mouse MI model. Using a lentiviral transduction system to overexpress Sestrin2 in polarized M1 and M2 macrophages, we revealed that Sestrin2 predominantly functioned on M1 rather than M2 macrophages. Sestrin2 overexpression suppressed pro-inflammatory response of M1 macrophages both *in vitro* and *in vivo*. Furthermore, in the mouse MI model with selective depletion of endogenous macrophages and adoptive transfer of exogenous Sestrin2-overexpressing macrophages, the anti-inflammatory and repair-promoting effect of Sestrin2-expressing macrophages was demonstrated. Taken together, our study indicates the importance of Sestrin2 for suppression of M1 macrophage-mediated cardiac inflammation after MI.

## Materials and Methods

### Mouse MI Model

The animal study was approved by Yangtze University of Medicine Animal Care and Use Committee and was performed in accordance with Yangtze University Guidelines for the Use of Animals. Male C57BL/6J and C57BL/6.SJL mice (8- to 10-week old) were ordered from the Animal Laboratory Center of Wuhan University. Mice were anesthetized with 2% halothane (Sigma-Aldrich) with 40% oxygen and followed by 0.5% halothane with 40% oxygen. Artificial ventilation (0.3 ml tidal volume, 120 breaths/min) was provided by tracheotomy. An 8.0 nylon surgical suture was used for ligation of the left coronary artery 1.0 mm distal from the tip of the left auricle. The left coronary artery was then occluded for 60 min before reperfusion for 3–7 days. The same surgical procedure was done on sham-operated mice, except that left coronary artery was not occluded.

### Cell Isolation

Cells were isolated from infarcted myocardium according to our previous method (14). Briefly, mouse hearts were rapidly excised and placed in ice-cold Krebs–Henseleit (KH) buffer containing (in g/L) 2 glucose, 0.141 MgSO_4_, 0.16 NaH_2_PO_4_, 0.35 KCl, 6.9 NaCl, 2.1 NaHCO_3_, 0.373 CaCl_2_, 1 NaN_3_ at pH 7.4. Subsequently, the aorta was cannulated with a 22-gage tubing adaptor and flushed with ice-cold KH buffer to remove residual cells in the coronary vasculature. The infarcted tissue were harvested and minced with fine scissors and placed into a cocktail of 0.25 mg/ml Liberase Blendzyme 3 (Roche Applied Science), 20 U/ml DNase I (Sigma-Aldrich), 10 mmol/L HEPES (Invitrogen), 0.1% sodium azide in HBSS with Ca^2+^ and Mg^2+^ (Invitrogen) and shaken at 37°C for 40 min. Cells were then passed through a 40-µm nylon mesh (BD Falcon), centrifuged for 10 min at 500 *g* at 4°C, and resuspended in 0.1% sodium azide solution in HBSS without Ca^2+^ and Mg^2+^. Isolated cells were kept on ice before further processing. In some experiments, infarcted tissues from 5 to 6 mice were pooled for cell isolation.

### Flow Cytometry Analysis

Anti-mouse antibodies were used: APC anti-CD3 (17A2), APC anti-B220 (RA3-6B2), APC anti-Gr1 (RB6-8C5), APC anti-CD90 (OX-7), FITC anti-CD11b (M1/70), PE anti-F4/80 (T45-2342), and PE-Cy7 anti-Ly-6C (AL-21) were purchased from BD Biosciences. PE-Cy5 anti-CD80 (16-10A1), PE-Cy7 anti-CD86 (GL1), and APC-eFluor^®^ 780 anti-CD45.1 (A20) were purchased from eBioscience. Biotin anti-CD163 (6E10.1G6) was purchased from Novus Biologicals. For cell surface staining, cells were incubated with corresponding antibodies in PBS for 15 min on ice before analysis on a BD LSRII flow cytometer. Dead cells were excluded with propidium iodide (2 µg/ml) staining. For phospho-flow assay, PE anti-phospho-4EBP1 (Thr36/45, clone # V3NTY24, eBioscience) was used to stain cells following the manufacturer’s instructions. All flow cytometry data were analyzed with Flowjo 7.6.1 software. Cell sorting was performed on a BD FACSAria cell sorter based on cell surface marker staining.

### Cell Culture, Lentiviral Transduction

Monocyte-derived macrophages were generated according to pervious reports (15–17). Briefly, normal mouse blood was taken into Falcon tubes with 2 mM EDTA. Spleens were collected from normal mice and pushed through 40-µm nylon meshes to prepare splenocyte suspension. The splenocyte suspension and blood were mixed before density gradient isolation with Ficoll-Paque™ PLUS (GE Healthcare), and mononuclear cells were collected and incubated in RPMI medium with 10% fetal calf serum, 4 mM l-glutamine, and penicillin/streptomycin. The cell density was 1 × 10^6^ cells/ml. 2 × 10^6^ cells were then seeded in each well of 6-well culture plates and were incubated overnight in a humid incubator with 5% CO_2_. Floating cells were then aspirated. Adherent cells were treated for 6 days either with 50 ng/ml GM-CSF and 10 ng/ml IFN-γ (for M1 polarization), or with 50 ng/ml M-CSF and 10 ng/ml IL-4 and 10 ng/ml IL-10 (for M2 polarization). All cytokines were purchased from R&D Systems.

For lipopolysaccharides (LPS) stimulation, polarized macrophages were gently washed with PBS once before digestion with 0.25% trypsin-EDTA for 5 min at 37^o^C. Cells were then plated at 1 × 10^5^ per well in 6-well plates in the presence or absence of 1 µg/ml LPS for 3 h. In some experiments, cells were pretreated with 2 µM MHY1485 (mTOR activator, Sigma-Aldrich) for 1 h before LPS treatment.

Lentiviral transduction of monocyte-derived macrophages was conducted according to previous successful reports (18–20). Briefly, polarized macrophages were gently washed with PBS once before digestion with 0.25% trypsin-EDTA for 5 min at 37^o^C. Cells were resuspended in growth media at the density of 5 × 10^6^ cells/ml. Three milliliters of cell suspension were added to each well of 6-well plates. Lentiviral particles (SESN2 lentiviral activation particles or control lentiviral particles, Santa Cruz Biotechnology) were diluted in growth medium containing 8 µg/ml polybrene (Sigma-Aldrich) and were added into each well at the MOI of 8. Cells were then incubated overnight with the lentiviral particles before wash once with PBS. Cells were then cultured in fresh growth medium in the presence of above cytokines for additional 5 days before further experiments. To monitor the lentiviral transduction efficiency, copGFP lentiviral particles containing GFP sequence (Santa Cruz Biotechnology) were transduced under the same condition as for SESN2 lentiviral activation particles. copGFP lentiviral particles has the same envelope proteins as SESN2 lentiviral activation particles.

### Macrophage Depletion and Adoptive Transfer

For selective depletion of monocytes/macrophages, each C57BL/6J mouse were injected i.v. with 0.1 ml of Clophosome^®^ (FormuMax Scientific) 24 h before MI. Exogenous macrophages were derived from C57BL/6.SJL mice and were labeled with carboxyfluorescein succinimidyl ester (CFSE, Thermofisher) or CellTrace Violet (Thermofisher) following the manufacturer’s instructions. Immediately before MI, 1 × 10^6^ or 4 × 10^6^ labeled macrophages in 200 µl of PBS were transferred into each C57BL/6J recipient mouse through retro-orbital injection.

### Enzyme Activity Assay

For assessment of proteolytic activity, 5 nmol of MMPsense-680 or Prosense-680 (both from Perkin Elmer) in 150 µl of PBS were retro-orbitally injected into each mouse 24 h before sacrifice. Fluorescence of each probe was evaluated on a BD LSRII flow cytometer.

### Histological Analysis

For quantification of infarct size, mice were anesthetized again. The heart was exposed and left coronary artery was ligated in the same spot. 0.3 ml of 1% Evans blue was perfused into the aorta to stain the heart for discrimination of the ischemic area from the non-ischemic area. The left ventricle of each heart was excised and was frozen and cut transversely into five slices of equal thickness followed by incubation in 1% triphenyltetrazolium chloride (TTC, sigma)/PBS at 37°C for 8 min and then were fixed in 10% formalin-PBS for 24 h. Fixed slices were then scanned, and ImageJ software (NIH) was used to measure the infarct area.

Collage deposition was analyzed by Masson staining. Briefly, hearts were excised and fixed with 10% formalin followed by dehydration and paraffin embedding. Six-micron sections were prepared and stained using Trichrome Stain (Masson) Kit (Sigma-Aldrich) following the vendor’s manual. The collagen fibers were quantified using collagen volume fraction (CVF), which is calculated as the ratio between the area of collagen staining and the area of the whole field. Five fields were randomly chosen and Image-pro plus 6.0 was used for the quantification.

### RNA Extraction, cDNA Synthesis, and Quantitative PCR (q-PCR)

Cellular or tissue RNAs were extracted using Trizol reagent (Invitrogen). cDNA synthesis was conducted with extracted RNAs and SuperScript^®^ III First-Strand Synthesis System (Invitrogen) according to the manufacturers’ instruction. q-PCR was conducted using Fast SYBR^®^ Green Master Mix (Invitrogen) on a 7300 q-PCR System (Invitrogen). Data were evaluated with 7300 system software. Primer sequences for each gene are listed in Table S1 in Supplementary Material. Relative abundance of gene expression was assessed using 2^−ΔΔCt^ method.

### Western Blot

The following primary antibodies were used: rabbit monoclonal anti-mTOR and anti-phospho-mTOR (Ser2448) were purchased from Cell Signaling Technology. Mouse monoclonal anti-β-actin was purchased from Santa Cruz Biotechnology. Rabbit monoclonal anti-Sestrin2 was purchased from Abcam. Membranes were developed with SuperSignal West Pico Chemiluminescent Substrate (Thermo Scientific) and were visualized and the optical density of the identified protein bands on membranes was analyzed using a Biospetrum 500 imaging system (UVP, LLC).

### Echocardiography

At day 14 after MI, mice were anesthetized with isoflurane inhalation. Left ventricle function was assessed by echocardiography with tracheal intubation. Echocardiographic data were collected by a HP/Philips Sonos 5500 ultrasound system with 15-MHz linear array transducer. Five consecutive cardiac cycles were used for each measurement. Left ventricle end-systolic diameter (LVESD) and end-diastolic diameter (LVEDD) were measured at end-diastole and end-systole, respectively. Percentage fractional shortening (%FS) and ejection fraction (EF) were calculated as: %FS = [(LVEDD − LVESD)/LVEDD] × 100; EF = 100 × [(LVEDD^3^ − LVESD^3^)/LVEDD^3^].

### Statistical Analysis

Data were analyzed and results were presented as mean ± SD. Student’s *t*-test or one-way ANOVA was used for comparison of mean between the groups, and *p* < 0.05 was considered significant.

## Results

### Cardiac Macrophages Express Sestrin2 in Infarcted Myocardium

**Figure 1:**
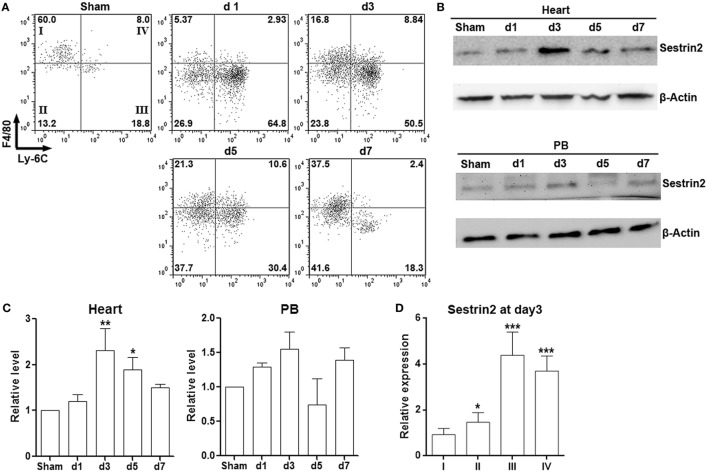
Sestrin2 is expressed in cardiac macrophages after myocardial infarction (MI). **(A)** Representative flow cytometry dot plots showing cardiac macrophage subpopulations in infarcted myocardium at day 1, day 3, day 5, and day 7 after MI. Numbers in the quadrants are proportions of corresponding subpopulations. Sham: Sham-operated animal. This is a representative of three independent experiments. **(B)** Sestrin2 protein levels in total cardiac macrophages (upper panel) and blood monocytes (lower panel). **(C)** Statistics for **(B)**. *N* = 3 per group. *p < 0.05; **p < 0.01; ***p < 0.001 compared with d1 group. **(D)** mRNA abundance of Sestrin2 in four cardiac macrophage subpopulations at day 3 after MI. *p < 0.05; **p < 0.01; ***p < 0.001 compared with subpopulation “I.”

To characterize cardiac macrophages in normal and ischemic myocardium, cells were isolated from infarcted area after ligature of the left anterior descending artery, as well as from the corresponding area in sham hearts. As shown in Figure S1 in Supplementary Material, a significant increase of CD3^−^B220^−^Gr-1^−^CD90^−^CD11b^+^ population, which represents macrophages, was observed in infarcted myocardium in comparison with sham control. Further analysis of expression of Ly-6C and F4/80 indicated that more than 50% of cardiac macrophages were pro-inflammatory F4/80^lo^Ly-6C^+^ cells in the acute phase of MI (post-MI day 1 to day 3). However, the phenotype of cardiac macrophages shifted toward F4/80^hi^Ly-6C^−^ cells from post-MI day 5 to day 7, suggesting the decrease of pro-inflammatory macrophages at the chronic stage of MI (Figure 1A). Our data were consistent with the previous phenotypic characterization of cardiac macrophages after MI (4). Interestingly, Sestrin2 expression was significantly enhanced in cardiac macrophages after MI and peaked at post-MI day 3 (Figures 1B,C). However, only a non-significant increase of Sestrin2 protein was seen in circulating monocytes after MI, suggesting that local factors rather than systemic factors were responsible for the increase of Sestrin2 in macrophages (Figures 1B,C). Moreover, careful analysis of Sestrin2 expression in F4/80^hi^ Ly-6C^−^, F4/80^lo^Ly-6C^−^, F4/80^lo^Ly-6C^+^, and F4/80^hi^Ly-6C^+^ subpopulations (subpopulation I–IV, respectively) revealed that Sestrin2 was predominantly expressed in Ly-6C^+^ macrophages at day 3 after MI (Figure 1D), suggesting that Sestrin2 was closely associated with the pro-inflammatory phenotype of cardiac macrophages. We also stained CD163 and CD80 to distinguish M1 and M2 macrophage subtypes. We found that about 50% of macrophages were CD163^-^CD80^+^ M1 macrophages, 30% were CD163^+^CD80^-^ M2 macrophages, 15% were CD163^−^CD80^−^ macrophages, and only 7% were CD163^+^CD80^+^ macrophages (Figure S2 in Supplementary Material). Sestrin 2 expression was upregulated in CD163^−^CD80^+^ and CD163^−^CD80^−^ macrophages, as compared with CD163^+^CD80^−^ macrophages (Figure S2 in Supplementary Material), suggesting that Sestrin 2 was mainly expressed in M1 macrophages.

### Sestrin2 Inhibits Pro-inflammatory Response of M1 Macrophages *In Vitro*

**Figure 2:**
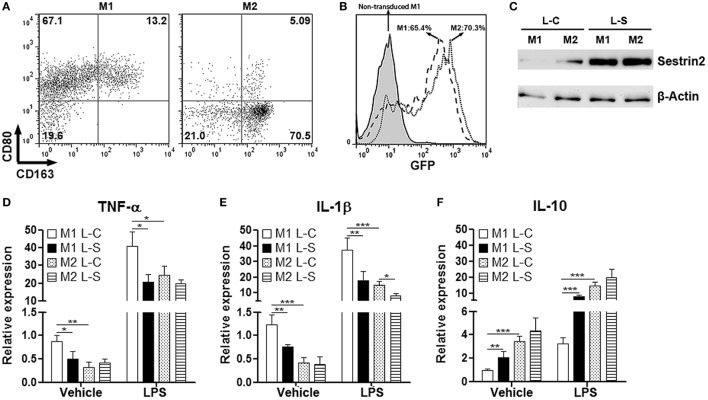
Ectopic Sestrin2 expression in M1 macrophages inhibits pro-inflammatory response *in vitro*. **(A)** Expression of CD80 and CD163 on *in vitro* cultured M1 and M2 monocyte-derived macrophages. Numbers in the quadrants are proportions of corresponding subpopulations. This is a representative of two independent experiments. **(B)** Lentiviral transduction efficiency of cultured macrophages is detected with flow cytometry. Note that here macrophages were not transduced with GFP-free SESN2 lentiviral activation particles. Instead, they were transduced by copGFP lentiviral particles containing GFP sequence. Numbers in the histogram are percentages of GFP^+^ cells. **(C)** Sestrin2 protein levels in M1 and M2 macrophages after lentiviral transduction. L-C: control lentivirus not inducing Sestrin2 expression. L-S: SESN2 lentiviral activation particles. This is a representative of three independent experiments. **(D–F)** mRNA abundance of TNF-α, IL-1β, and IL-10 in lentivirus-transduced M1 and M2 macrophages with or without lipopolysaccharide (LPS) stimulation. M1 L-C: M1 macrophages transduced with control lentivirus. M1 L-S: M1 macrophages transduced with SESN2 lentiviral activation particles. M2 L-C: M2 macrophages transduced with control lentivirus. M2 L-S: M2 macrophages transduced with SESN2 lentiviral activation particles. *N* = 6 per group. *p < 0.05; **p < 0.01; ***p < 0.001.

To explore the effects of Sestrin2 on macrophages, we cultured monocyte-derived macrophages under M1 (with GM-CSF and IFN-γ) or M2 (with M-CSF and IL-4) polarization condition. Flow cytometry analysis indicated that nearly 70% of cultured cells were CD80^+^CD163^lo/−^ in the presence of GM-CSF and IFN-γ, suggesting the majority was M1 macrophages (Figure 2A). However, in the presence of M-CSF and IL-4, 70% of macrophages were CD80^−^CD163^+^, suggesting the successful M2 polarization (Figure 2A). These cells were then transduced with SESN2 lentiviral activation particles to induce ectopic expression of Sestrin2. Flow cytometry analysis demonstrated that more than 65% of either M1 or M2 macrophages were successfully transduced with lentivirus (Figure 2B). And the ectopic Sestrin2 expression in either M1 or M2 macrophages was verified by Western blot (Figure 2C). We then determined the cytokine production in macrophages with or without LPS stimulation. As shown in Figures 2D,E, ectopic Sestrin2 expression alleviated TNF-α and IL-1β mRNA levels in M1 macrophages in the presence or absence of LPS. However, in M2 macrophages, ectopic Sestrin2 expression only moderately reduced IL-1β level, while TNF-α production was not significantly altered. In addition, Sestrin2 overexpression promoted IL-10 mRNA abundance only in M1 macrophages (Figure 2F).

### Sestrin2 Suppresses Pro-inflammatory Response of M1 Macrophages *In Vivo*

**Figure 3:**
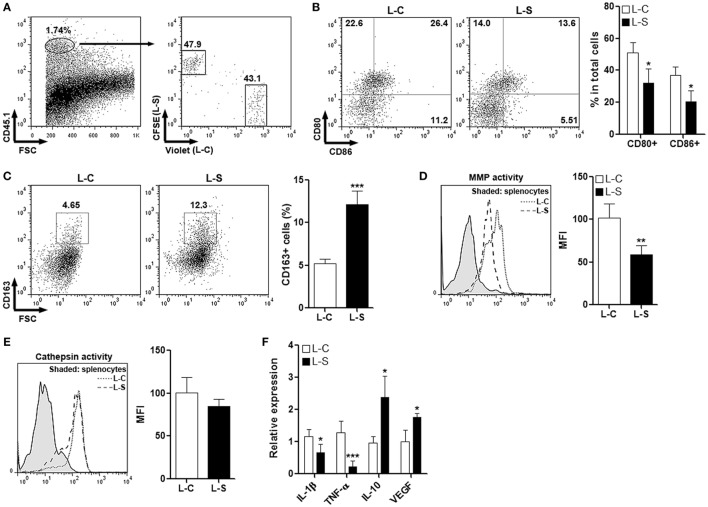
Ectopic Sestrin2 expression in M1 macrophages inhibits pro-inflammatory response *in vivo*. **(A)** Detection of adoptively transferred M1 macrophages in infarcted myocardium at day 3 after myocardial infarction. Note that M1 macrophages were derived from C57BL/6.SJL mice, transduced with lentivirus (L-C or L-S, respectively), and labeled with CellTrace Violet or carboxyfluorescein succinimidyl ester (CFSE) before adoptive transfer. Numbers in the plots are proportions of gated cells populations. **(B)** Expression of CD80 and CD86 on transferred M1 macrophages. Left panel: representative dot plots. Right panel: statistics. L-C: M1 macrophages transduced with control lentivirus. L-S: M1 macrophages transduced with SESN2 lentiviral activation particles. **(C)** Expression of CD163 on transferred M1 macrophages. Left panel: representative dot plots. Right panel: statistics. **(D,E)** MMP activity **(D)** and Cathepsin activity **(E)** in transferred M1 macrophages. Left panel: representative histograms. Right panel: statistics. **(F)** mRNA abundance of indicated cytokines in transferred M1 macrophages. *N* = 4–6 per group. *p < 0.05; **p < 0.01; ***p < 0.001 in comparison with L-C.

To test the effect of Sestrin2 on M1 macrophages *in vivo*, monocytes were collected from C57BL/6.SJL mouse blood and spleens, induced to be M1 macrophages, and transduced with lentiviruses. Macrophages transduced with control lentivirus (L-C) and SESN2 lentiviral activation particles (L-S) were labeled with CellTrace Violet and CFSE, respectively. These two macrophage populations were then mixed at the ratio of 1:1 and adoptively injected into recipient C57BL/6 mice immediately before MI was conducted. Three days after MI, cardiac macrophages were analyzed. As shown in Figure 3A, exogenous CD45.1^+^ macrophages were present in infarcted myocardium, and the ratio of Violet-positive cells vs CFSE-positive cells was close to 1:1, suggesting that Sestrin2 overexpression did not influence recruitment of M1 macrophages into infarcted myocardium. Analysis of macrophage surface markers indicated that Sestrin2 overexpression down-regulated CD80 and CD86 expression while upregulating CD163 expression on these macrophages (Figures 3B,C), suggesting that Sestrin2 induced a phenotypic shift from M1 type toward M2 type. Additionally, Sestrin2 overexpression impaired the MMP activity of macrophages, whereas the cathepsin activity was not changed (Figures 3D,E). Moreover, Sestrin2 overexpression decreased production of pro-inflammatory cytokines such as TNF-α and IL-1β, and increased repair-promoting cytokines like IL-10 and VEGF (Figure 3F).

### Sestrin2 Promotes M2 Macrophage Function

**Figure 4:**
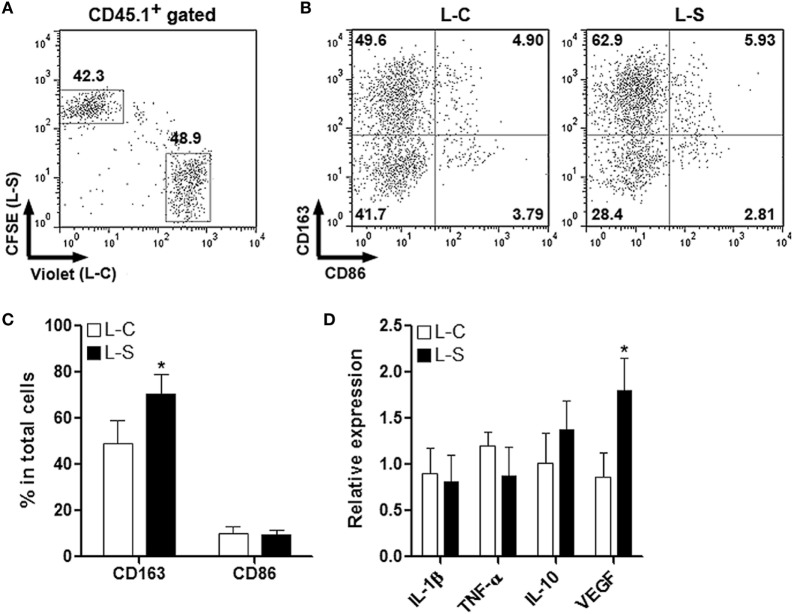
Ectopic Sestrin2 expression in M2 macrophages has limited effects *in vivo*. **(A)** Detection of adoptively transferred M2 macrophages in infarcted myocardium at day 3 after myocardial infarction. Note that M2 macrophages were derived from C57BL/6.SJL mice, transduced with lentivirus (L-C or L-S, respectively), and labeled with CellTrace Violet or carboxyfluorescein succinimidyl ester (CFSE) before adoptive transfer. Numbers in the plots are proportions of gated cells populations. **(B,C)** Expression of CD163 and CD86 on transferred M2 macrophages. **(B)** Representative dot plots. **(C)** Statistics. L-C: M2 macrophages transduced with control lentivirus. L-S: M2 macrophages transduced with SESN2 lentiviral activation particles. **(D)** mRNA abundance of indicated cytokines in transferred M2 macrophages. *N* = 3 per group. *p < 0.05 in comparison with L-C.

To test the effect of Sestrin2 on M2 macrophages *in vivo*, monocytes were collected from C57BL/6.SJL mouse blood and spleens, induced to be M2 macrophages, and transduced with lentiviruses as done for M1 macrophages. M2 macrophages were then labeled and adoptively transferred the same way as for M1 macrophages. Similarly, Sestrin2 overexpression did not profoundly impact the recruitment of M2 macrophages into infarcted myocardium (Figure 4A). In addition, M2 macrophages transduced with L-S had higher CD163 expression, suggesting that Sestrin2 maintained or enhanced M2 polarization (Figures 4B,C). However, Sestrin2 overexpression did not considerably alter the mRNA levels of TNF-α, IL-1β, and IL-10, whereas VEGF expression was moderately increased (Figure 4D). Taken together, our data imply that the effects of Sestrin2 on M2 macrophage phenotype and function are limited.

### Sestrin2 Expression in M1 Macrophages Mitigates Postischemic Myocardial Inflammation

**Figure 5:**
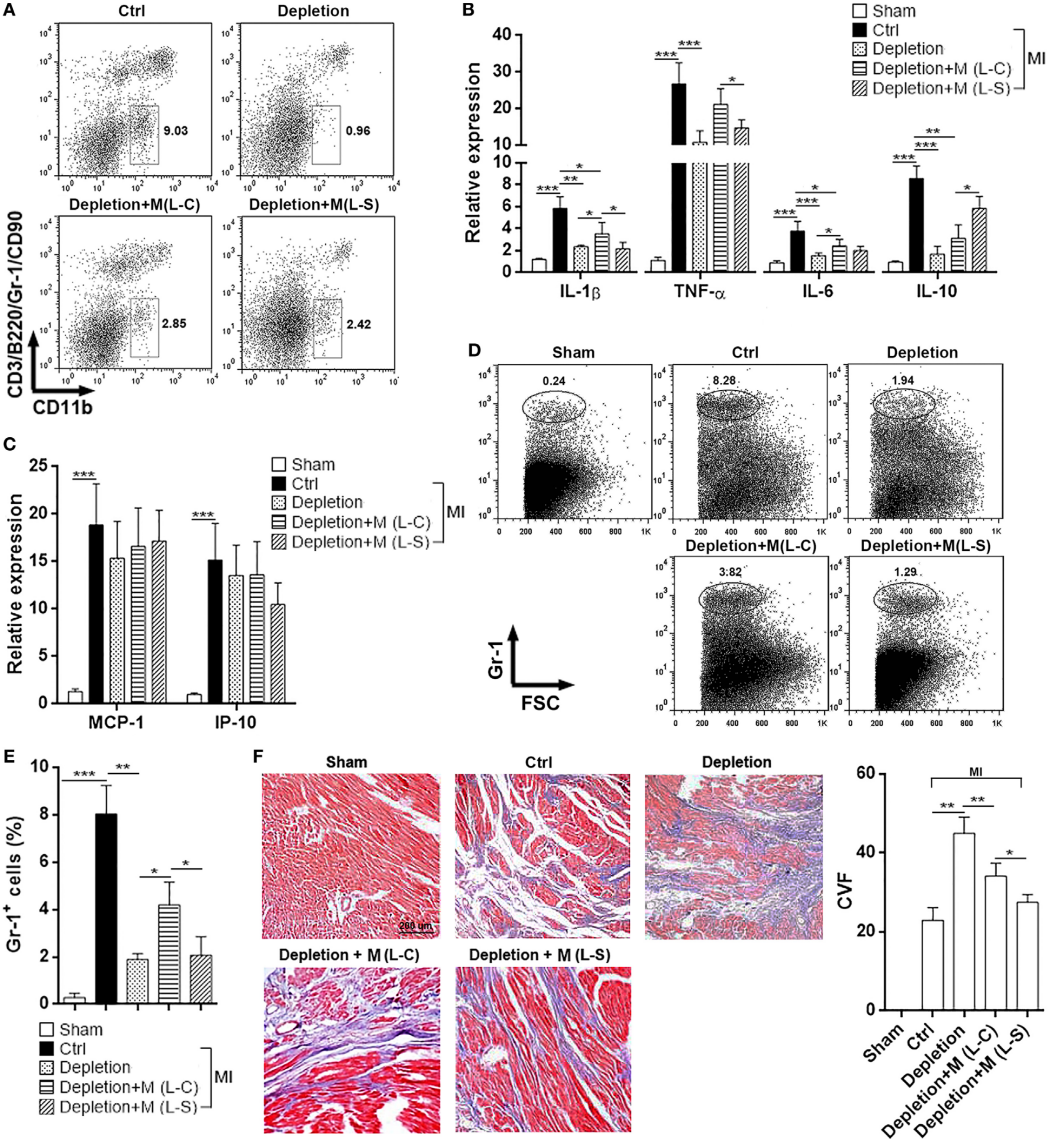
Ectopic Sestrin2 expression in M1 macrophages attenuates post-MI inflammation and favors tissue repair in hearts. **(A)** Macrophage abundance in infarcted myocardium at day 3 after myocardial infarction (MI) (day 4 after Clophosome^®^ treatment). Ctrl: un-pretreated mice. Depletion: Clophosome^®^ injection. Depletion + M (L-C): Clophosome^®^ injection followed by transfer of M1 macrophages transduced with control lentivirus. Depletion + M (L-S): Clophosome^®^ injection followed by transfer of M1 macrophages transduced with SESN2 lentiviral activation particles. Numbers in the plots are proportions of gated cells populations. This is a representative of two independent experiments. **(B,C)** mRNA abundance of indicated cytokines and chemokines in infarcted myocardium. **(D,E)** Percentage of Gr-1^+^ neutrophils in the whole cardiac cells at day 3 after MI. Representative flow cytometry dot plots are shown in **(D)**, and statistics is shown in **(E)**. **(F)** Masson staining at day 7 after MI. Left panel: representative images. Right panel: statistics of collagen volume fraction (CVF). *N* = 4–5 per group. *p < 0.05; **p < 0.01; ***p < 0.001.

Because above data suggest the significant role of Sestrin2 in modulating M1 macrophage response, we then determined the effect of Sestrin2-overexpressing M1 macrophages on the postischemic myocardial inflammation. Endogenous macrophages were temporarily depleted using Clophosome^®^, and exogenous Sestrin2-overexpressing M1 macrophages were adoptively transferred into mice immediately before MI. As shown in Figure S3 in Supplementary Material, over 90% of endogenous blood monocytes were depleted 24 h after administration of Clophosome^®^. Similar depletion of endogenous cardiac macrophages was also observed in Clophosome^®^-injected mice (Figure 5A). Adoptive transfer of exogenous macrophages partially restored cardiac macrophage abundance in Clophosome^®^-injected mice (Figure 5A). Analysis of cytokine expression in infarcted myocardium revealed that MI itself profoundly induced expression of pro-inflammatory cytokines like TNF-α, IL-1β, and IL-6 (Figure 5B). Noteworthy, compared with mice receiving L-C-transduced macrophages, mice receiving L-S-transduced macrophages had lower expression of these cytokines in infarcted myocardium (Figure 5B). In addition, expression of anti-inflammatory IL-10 was higher in the myocardium of mice receiving L-S-transduced macrophages, in comparison with mice receiving L-C-transduced macrophages (Figure 5B). However, the expression of chemokines MCP-1 and IP-10, which have been shown to mediate leukocyte recruitment, was not significantly changed between mice receiving L-C-transduced macrophages and mice receiving L-S-transduced macrophages (Figure 5C). To further characterize the post-MI myocardial inflammation, the infiltrating neutrophils were quantified in the hearts. As shown in Figures 5D,E, very little neutrophils were recovered from sham-operated hearts, while substantial amount of neutrophils was found in infarcted hearts. Depletion of macrophages decreased infiltrating neutrophils. Furthermore, in comparison with mice receiving L-C-transduced macrophages, the mice receiving L-S-transduced macrophages had less infiltrating neutrophils in infarcted hearts, suggesting that Sestrin2-overexpressing macrophages induced myocardial inflammation to a less extent. In addition, Masson staining showed that L-S-transduced macrophages caused less collagen staining in infarcted myocardium, as compared with L-C-transduced macrophages (Figure 5F), suggesting that Sestrin2-expressing macrophages might result in a scar with low collagen content.

### Sestrin2 Suppresses mOTRC1 Signal Pathway in M1 Macrophages

**Figure 6:**
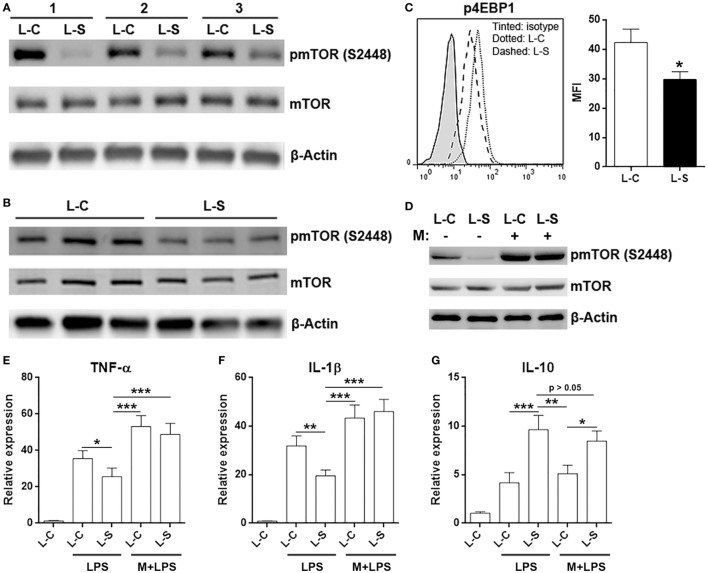
Sestrin2 inhibits mTORC1 signal pathway in M1 macrophages. **(A)** mTOR phosphorylation in transferred M1 macrophages. M1 macrophages were transduced with lentivirus and transferred into recipient mice as in Figure 5. These macrophages were then sorted using flow cytometry from infarcted myocardium at day 3 after myocardial infarction (MI) for detection of mTORC1 activation status. L-C: M1 macrophages transduced with control lentivirus. L-S: M1 macrophages transduced with SESN2 lentiviral activation particles. #1 to #3 indicate three mice. **(B)** mTOR phosphorylation in *in vitro* cultured M1 macrophages after lentiviral transduction. **(C)** 4EBP1 phosphorylation in transferred M1 macrophages at day 3 after MI. *N* = 3 per group. **(D)** mTOR phosphorylation in *in vitro* cultured M1 macrophages in the presence or absence of 1-h MHY1485 treatment. L-C: M1 macrophages transduced with control lentivirus. L-S: M1 macrophages transduced with SESN2 lentiviral activation particles. This is a representative image of two independent experiments. **(E–G)** mRNA abundance of indicated cytokines in *in vitro* cultured M1 macrophages. LPS: LPS stimulation. M + LPS: MHY1485 pretreatment followed by LPS stimulation. *N* = 5 per group. *p < 0.05; **p < 0.01; ***p < 0.001.

It has been previously shown that Sestrin2 inhibits mTORC1 signal pathway (21, 22), and mTORC1 is crucial for M1 macrophage polarization and function (23, 24). Therefore, we checked activation status of mTORC1 signal pathway in adoptively transferred L-S-transduced macrophages and L-C-transduced macrophages. Interestingly, activating phosphorylation of mTOR at serine 2448 was decreased in L-S-transduced macrophages (Figure 6A). This was also seen in *in vitro* cultured L-S-transduced macrophages (Figure 6B). Consistently, phosphorylation of 4EBP1 in L-S-transduced macrophages was lower than that in L-C-transduced macrophages (Figure 6C). To further confirm the role of mTORC1 signaling in Sestrin2-induced changes, we pretreated L-C or L-S-transduced macrophages with an mTOR activator, MHY1485, and then stimulated macrophages with LPS. As shown in Figure 6D, MHY1485 increased mTOR phosphorylation in both L-C-transduced and L-S-transduced macrophages, in comparison with non-treated L-C-transduced macrophages. In addition, MHY1485 pretreatment promoted TNF-α and IL-1β expression in in both L-C-transduced and L-S-transduced macrophages, suggesting that forced mTORC1 activation counteracted Sestrin2’s effect and enhanced inflammatory response of macrophages (Figures 6E,F). However, IL-10 expression was not dramatically impacted by MHY1485 pretreatment, suggesting that mTORC1 signaling might be irrelevant to IL-10 production (Figure 6G). Thus, Sestrin2-induced upregulation of IL-10 might not be related to mTORC1 signaling.

### Adoptive Transfer of Sestrin2-Overexpressing Macrophages Does Not Improve Heart Function

**Figure 7:**
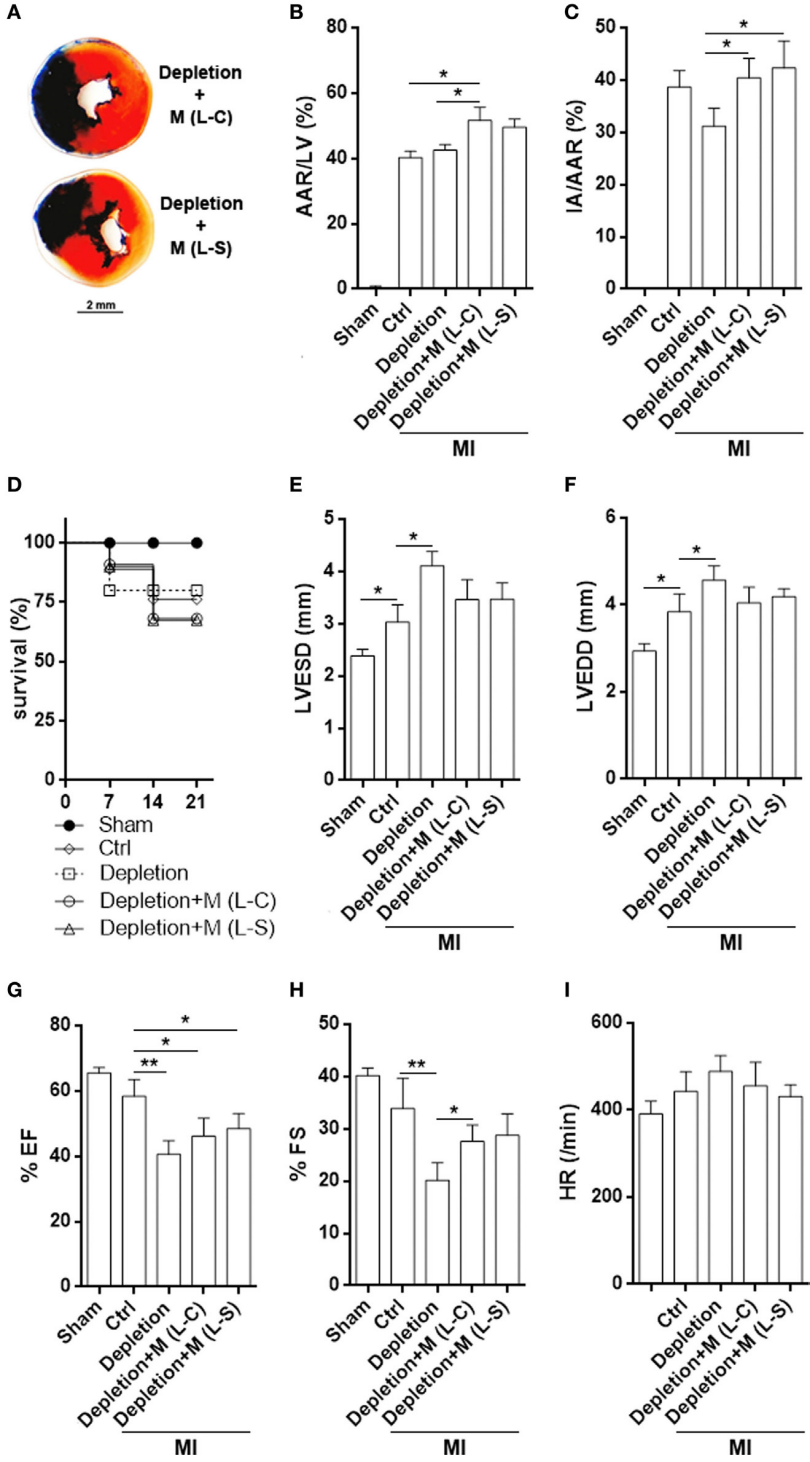
Infarct size and left ventricle function after myocardial infarction (MI). **(A)** Representative triphenyltetrazolium chloride staining image. **(B,C)** Measurement of area at risk and infarct area. Ctrl: un-pretreated mice. Depletion: Clophosome^®^ injection. Depletion + M (L-C): Clophosome^®^ injection followed by transfer of M1 macrophages transduced with control lentivirus. Depletion + M (L-S): Clophosome^®^ injection followed by transfer of M1 macrophages transduced with SESN2 lentiviral activation particles. AAR/LV: area at risk volume relative to left ventricle volume. IA/AAR: infarct area relative to area at risk. *N* = 6 per group. **(D)** Mouse survival after MI. **(E–I)** Evaluation of left ventricle end-systolic diameter (LVESD), LVEDD, FS, ejection fraction (EF), and heart rate by echocardiography 2 weeks after MI. *N* = 5–6 per group. *p < 0.05; **p < 0.01.

Although post-MI myocardial inflammation was moderately mitigated by L-S-transduced macrophages, TTC staining demonstrated comparable infarct size in mice receiving L-C-transduced macrophages and mice receiving L-S-transduced macrophages (Figures 7A–C). Macrophage depletion caused a trend of decrease in infarction size (Figure 7C). Meanwhile, the survival rate was also similar between mice receiving L-S-transduced macrophages and those receiving L-C-transduced macrophages (Figure 7D). Evaluation of left ventricle function revealed that depletion of macrophages increased LVESD and LVEDD after MI in comparison with the control group. Correspondingly, FS and EF were decreased when macrophages were depleted (Figures 7E–I). Transfer of exogenous macrophages caused partial but insignificant decrease in LVESD and LVEDD, as well as increase in FS. However, LVESD, LVEDD, FS, EF, and heart rates were comparable between mice receiving L-S-transduced macrophages and mice receiving L-C-transduced macrophages (Figures 7E–I).

## Discussion

Macrophages, in addition to their effects on innate immunity and tissue homeostasis, play a crucial role in the MI-induced pathological processes. In particular, it is hypothesized that cardiac macrophages maintain heart hemostasis under normal condition and contribute to wound healing, regeneration, and tissue remodeling in cardiac diseases (25). Therefore, understanding how macrophage functions are modulated in infarcted myocardium is of great importance for developing therapeutic interventions for MI.

In this study, we characterize the role of Sestrin2 in regulating macrophage-mediated inflammatory response after MI. Interestingly, there seemed to be a dynamic expression of Sestrin2 in cardiac macrophages, while Sestrin2 expression in circulating monocytes remained low. It is noteworthy that Sestrin2 expression peaked at day 3 and then decreased until day 7 after MI, suggesting that Sestrin2 might be associated with transition from acute inflammation to chronic inflammation. Furthermore, Ly-6C^+^ pro-inflammatory macrophages expressed higher Sestrin2 than Ly-6C^−^ macrophages, suggesting Sestrin2 might be related to inflammatory response of cardiac macrophages. However, the factors that upregulated Sestrin2 expression have not been identified. A previous research suggests that NO and hypoxia upregulate Sestrin2 by HIF-1-dependent pathway in macrophages (26). Indeed, former studies of MI induced in rats showed that HIF-1α and -2α were upregulated in macrophages in infarcted tissue (27). Other mechanisms, such as mitochondria-dependent ROS production (28), Toll-like receptor-mediated AP-1, and Nrf2 signaling (29), have also been shown to induce Sestrin2 expression in macrophages or other cell types. Hence, our lab will test the involvement of these signal pathways in Sestrin2 expression in cardiac macrophages after MI.

To examine the effect of Sestrin2 on cardiac macrophages *in vivo*, we applied lentiviral transduction to induce overexpression of Sestrin2 in either M1 or M2 macrophages. We did so because M1 and M2 macrophages coexist in infarcted myocardium (4, 5), but the effects of Sestrin2 on these polarized macrophages remain unknown. It is surprising that Sestrin2 overexpression markedly suppressed LPS-induced inflammatory response of M1 macrophages, but could only weakly regulate the same response of M2 macrophages. This phenomenon suggests that Sestrin2 might function on M1 and M2 macrophages through distinct signal pathways, or the responsiveness of macrophage subsets to Sestrin2 expression are not uniform. Consistently, Sestrin2 overexpression profoundly suppressed M1 macrophage-mediated inflammation in infarcted myocardium. The attenuation of post-MI inflammation could reduce secondary tissue damage and therefore be beneficial for tissue repair and regeneration.

Notably, Sestrin2 overexpression seemed to induce a moderate shift of M1 macrophages toward M2 macrophages. Previous research has discovered various signaling molecules that impact macrophage differentiation, including interferon regulatory factor (IRF) and signal transducers and activators of transcription (STAT) (30). It will be therefore interesting to check whether Sestrin2 impacts the expression or activation of these molecules and corresponding signal pathways. For example, Sestrin2 might downregulate expression of IRF5 which is critical for M1 polarization. Furthermore, perhaps Sestrin2 could not change the expression of STAT6 or IRF4 which are both crucial for M2 polarization, so it hardly alters M2 macrophage function. Another intriguing question is that if Sestrin2 functions differentially on individual M2 subset. It is now considered that the M2 macrophages involve distinct macrophage subsets, such as M2a, M2b, M2c, and M2d, according to their gene expression profiles (31). Unfortunately, whether these subsets are present in infarcted myocardium remains unknown. Our group is focusing on detection and delineation of M2 subsets after MI and hope to disclose the effect of Sestrin2 on each individual subset.

mTORC1 signaling has been proven to be critical for macrophage polarization and function (23, 24, 32, 33). Constitutive mTORC1 activity impairs M2 polarization while increasing inflammatory responses to pro-inflammatory stimuli (23). Myeloid-specific deletion of TSC1 enhances M1 type response and subsequently induces inflammatory diseases in mice (34). Interestingly, Sestrin2 has long been known to inhibit mTORC1 signaling and promote AMPK signaling (21, 22, 35). Consistently, our data also showed lower phosphorylation of mTOR and 4EBP1 in Sestrin2-overexpressing M1 macrophages both *in vitro* and *in vivo*. Therefore, the anti-inflammatory effect of Sestrin2 in M1 macrophages might be attributed to the suppression of mTORC1 signaling. However, since we have not observed significant effect of Sestrin2 in M2 macrophages, we did not check the activation status of mTORC1 signaling in M2 macrophages in current study. It is possible that Sestrin2 has no or different regulatory effects on mTORC1 signaling in M2 macrophages.

Although Sestrin2-overexpressing macrophages moderately mitigated post-MI myocardial inflammation, we did not observe significant changes of infarct size or heart function. This might be due to the limited amount of transferred macrophages. It would be possible to see the significant changes if more Sestrin2-overexpressing macrophages were present. Therefore, it will be absolutely necessary to use Sestrin2 transgenic or deficient mouse strains for future study to elucidate the beneficial effect of Sestrin2.

Taken together, our study indicates the importance of Sestrin2 to suppression of macrophage-mediated cardiac inflammation after MI.

## Ethics Statement

All animal experiments were conducted in compliance with institutional guidelines and Yangtze University Guidelines for the Use of Animals. All animal procedures were approved by Yangtze University of Medicine Animal Care and Use Committee.

## Author Contributions

KY conducted most experiments and analyzed the data; YZ performed mouse MI model; CX performed Western blot assay; SH conducted lentiviral transduction; DL designed the study and wrote the manuscript. All authors read and approved the final manuscript.

## Conflict of Interest Statement

The authors declare that the research was conducted in the absence of any commercial or financial relationships that could be construed as a potential conflict of interest.
