# Supplementary material for: Sestrin2 Suppresses Classically Activated Macrophages-Mediated Inflammatory Response in Myocardial Infarction through Inhibition of mTORC1 Signaling
Source: Front Immunol. 2017 Jun 30;8:728. doi: 10.3389/fimmu.2017.00728 (PMC5491632; doi:10.3389/fimmu.2017.00728)
Supplement: Supplementary file 1 [file Data_Sheet_1.docx]

**Supplemental table 1.**

**Primer sequences of q-PCR**

| Name | Forward (5’ to 3’) | Reverse (5’ to 3’) |
| --- | --- | --- |
| β-actin | AGAGGGAAATCGTGCGTGAC | CAATAGTGATGACCTGGCCGT |
| IL-10 | GATGCCTTCAGCAGAGTGAA | GCAACCCAGGTAACCCTTAAA |
| TGF-β | TGACGTCACTGGAGTTGTACGG | GGTTCATGTCATGGATGGTGC |
| Sestrin2 | ACACCATGATCGTAGCGGAC | CACTGGAATGAAAGCGCTGG |
| MCP-1 | GCTCAGCCAGATGCAGTTAA | TCTTGAGCTTGGTGACAAAAACT |
| IP-10 | CCAGTGAGAATGAGGGCCATA | CTCAACACGTGGGCAGGA T |
| TNF-α | GCCTCTTCTCATTCCTGCTTG | CTGATGAGAGGGAGGCCATT |
| IL-1β | TGTCTGACCCATGTGAGCTG | GCCACAGGGATTTTGTCGTT |
| IL-6 | ACTTCACAAGTCGGAGGCTT | TTCTGACAGTGCATCATCGCT |
| VEGF | ACTGGACCCTGGCTTTACTG | GCTTCGCTGGTAGACATCCA |

**Supplemental figures**


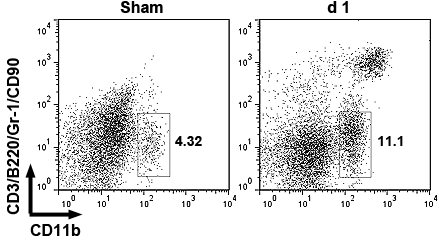


**Supplemental figure 1. Flow cytometry gating strategy for cardiac macrophages at sham-operated heart and infarcted myocardium at day 1 after MI.** Numbers in the plots are proportions of gated populations.


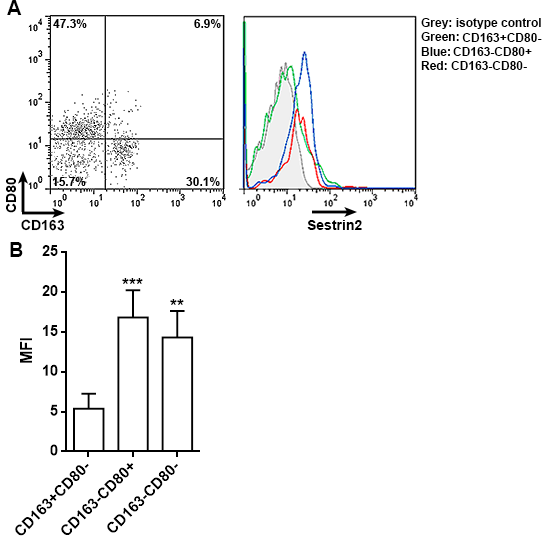


**Supplemental figure 2. Expression of Sestrin2 in macrophage subpopulations based on CD163 and CD80 staining.** At day 3 post MI, CD3^-^B220^-^Gr-1^-^CD90^-^CD11b^+^ macrophages in isolated cardiac cells, as shown in **supplemental figure 1**, were further stained with anti-CD80 and anti-CD163 antibodies, followed by intracellular staining with rabbit anti-Sestrin2 polyclonal antibody (MyBioSource.com) and Alexa Fluor® 790 Goat anti-Rabbit IgG (Thermo Fisher Scientific). **(A)** Representative dot plot of macrophage subpopulations based on CD163 and CD80 staining (left) and histograms of Sestrin2 staining in each subpopulation (right). Note that CD163^+^CD80^+^ macrophages were not shown in the histograms due to their limited cellular abundance. **(B)** Statistics for the mean fluorescent intensity of Sestrin2 in each subpopulation. **, *p*<0.01; ***, *p*<0.001 in comparison to CD163^+^CD80^-^ macrophages.


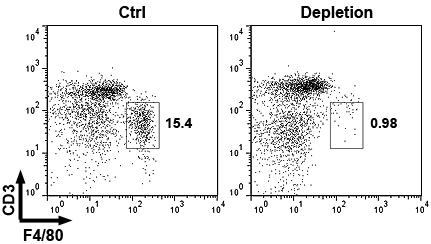


**Supplemental figure 3. Depletion of endogenous blood monocytes 24 h after administration of Clophosome®.** Peripheral blood mononuclear cells were separated and harvested after gradient isolation using iodixanol OptiPrep (Axis-Shield) according to the manufacturer’s instructions. Then cells were stained with APC anti-CD3 (17A2) antibody and PE anti-F4/80 (T45-2342) antibody followed by flow cytometry analysis. Numbers in the plots are proportions of gated populations. Ctrl: vehicle injection. Depletion: Clophosome® injection.
